# Pancreatic metastases originating from uterine leiomyosarcoma: a case report

**DOI:** 10.1186/1477-7819-12-405

**Published:** 2014-12-30

**Authors:** Simona Olimpia Dima, Nicolae Bacalbasa, Mihai Adrian Eftimie, Irinel Popescu

**Affiliations:** “Dan Setlacec” Center of General Surgery and Liver Transplantation, Fundeni Clinical Institute, Soseaua Fundeni 258, Bucharest, 022328 Romania; “Carol Davila” University of Medicine and Pharmacy, Bulevardul Eroii Sanitari 8, Bucharest, 050474 Romania

**Keywords:** Pancreatic metastases, Resectable oligometastases, Uterine leiomyosarcoma

## Abstract

In this report, we describe the case of a 67-year-old woman with metastatic pancreatic uterine leiomyosarcoma. She underwent a total hysterectomy and adnexectomy in December 2009. The resected uterine specimen was characterized as a leiomyosarcoma. The patient was free of disease until November 2010, when three pulmonary tumoral lesions detected by follow-up chest computed tomography were diagnosed as metastatic lesions. Wedge resections and enucleoresection of the pulmonary tumoral nodules were performed, and the patient received adjuvant chemotherapy. Ten months after the lung resection, an abdominal examination showed two tumoral masses in the pancreas and no extrapancreatic recurrence. In April 2014, a pylorus-preserving pancreaticoduodenectomy was performed. To date, the patient is alive, without any evidence of recurrence, and she has received chemotherapy. Surgery can be considered in cases in which the pancreas is a unique metastatic site or even in cases with resectable oligometastases.

## Background

Uterine leiomyosarcoma (LMS) is a rare malignancy with high metastatic potential. Patients with metastatic or recurrent disease have a poor prognosis with limited treatment options. The 5-year survival rates are 53% in patients with stage I uterine LMS and 8% in those with stages II through IV disease [1, 2]. The most common sites of metastatic LMS are the lung, the peritoneal cavity and the liver [3, 4]. A case of pancreatic metastasis from a uterine LMS is rare [5, 6]. Ogura *et al*. identified, in a literature review, 25 cases of LMS metastases to the pancreas, of which 7 cases were from a primary uterine tumor [7]. In this report, we describe a case of a patient with metastatic pancreatic uterine LMS.

## Case presentation

**Figure 1:**
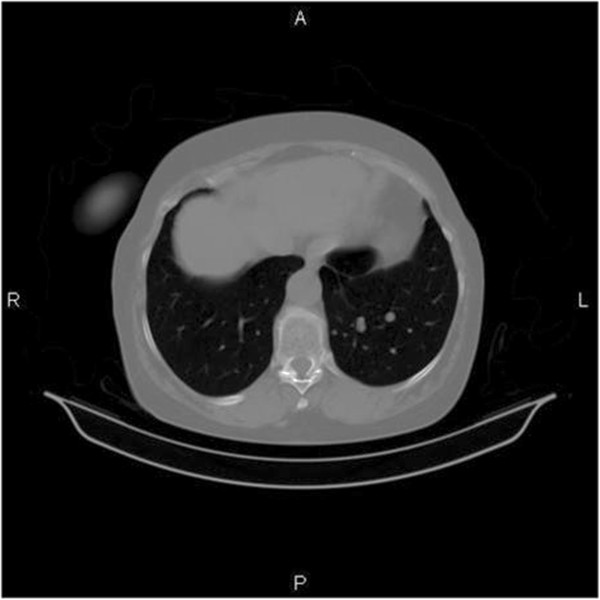
**Chest computed tomography.** The pulmonary nodular tumor invasion.

A 67-year-old womanwith a medical history of hypertension and hyperthyroidism presented to our hospital with postmenopausal vaginal bleeding in 2009. A transvaginal ultrasound showed a tumoral mass in the uterine wall that was initially interpreted as a leiomyoma. In December 2009, she underwent a total hysterectomy and bilateral adnexectomy. The surgically resected uterine specimen was characterized as a LMS (pT1c) with immunohistochemistry results positive for smooth muscle actin (SMA) and desmin and a Ki-67 level of 30%. No adjuvant radiochemotherapy was administered, but a follow-up examination every 6 months was recommended. The patient was free of disease until November 2010, when three pulmonary tumoral lesions 7 mm, 8.34-mm and 5 mm in size, respectively, in the inferior lobe of the left lung were detected by follow-up chest computed tomography (CT) (Figure 1). To clarify the differential diagnosis between a primary lung tumor and metastatic disease, the multidisciplinary panel decided to perform surgery.

**Figure 2:**
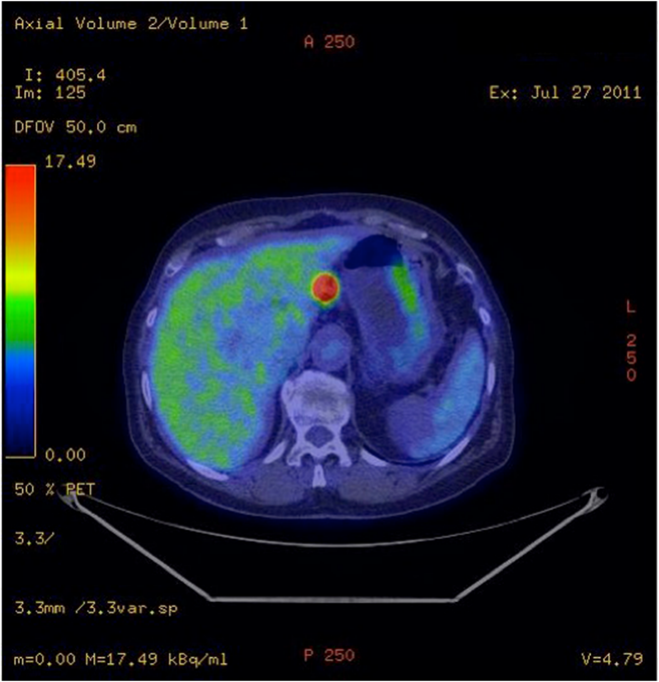
**Positron emission tomography/computed tomography revealed a mass in the pancreatic head with metabolic activity.**

Wedge resections and enucleoresection of the lung tumoral nodules were performed in November 2011. The histopathological examination of the resected lung specimen revealed the presence of spindle-shaped cells with a high mitotic count. The immunohistochemistry was positive for SMA and desmin and negative for CD10, MNF116 antibody and estrogen receptor/progesterone receptor, and her Ki-67 level was 30%, thus clarifying the diagnosis of a metastatic LMS. Postoperatively, the patient received chemotherapy consisting of one cycle of Epirubicin 150 mg + Cisplatin (CDDP)100 mg and gemcitabine + vinorelbine (six cycles).The regular follow-up CT scan obtained in July 2011, 10 months after the lung resection, showed two tumoral masses in the pancreas at the junction of the head and body. Whole-body positron emission tomography (PET) performed in September 2011 (Figure 2) showed heterogeneous enhancement at the level of the pancreatic head with metabolic activity, indicating malignant potential of the lesions, and no evidence of extrapancreatic metastatic lesions.

The oncological context and the multiplicity of the pancreatic lesions following the metastatic pulmonary disseminations oriented the diagnosis towards pancreatic metastases rather than a primary pancreatic tumor. In terms of the biohumoral markers, the carcinoembryonic antigen (CEA) and carbohydrate antigen (CA 19-9) levels were as follows: 2 ng/ml; CA 19-9, 14 U/ml; however, an above upper limit level of chromogranin A could have allowed a differential diagnosis of a primary pancreatic neuroendocrine tumor.

**Figure 3:**
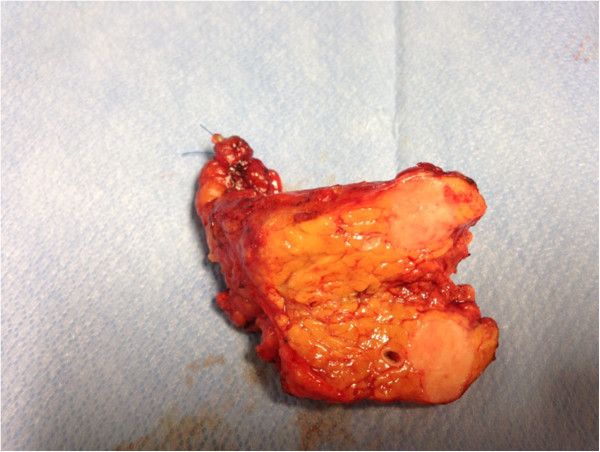
**Surgical specimen from the pancreaticoduodenectomy.**

Because of the previous episode of lung metastases, which suggested systemic dissemination, probably with other occult metastatic lesions, the oncological panel decided on systemic chemotherapy. In September 2011, the patient was switched to a combination of gemcitabine (1,800 mg/m^2^) plus dacarbazine (500 mg/m^2^). PET/CT performed in December 2013 showed a slight increase in the size of the pancreatic lesions and no signs of extrapancreatic recurrence. Because acquired chemoresistance was suspected and because the disease was apparently limited to the pancreas, a decision was made to perform a pancreatic resection. In April 2014, a pylorus-preserving pancreaticoduodenectomy was performed (Figure 3). Microscopically, the surgically resected pancreatic specimen showed features similar to those of the uterine LMS. The immunohistochemical studies showed that the neoplastic cells were positive for SMA and desmin and negative for chromogranin and synaptophysin.

The patient’s postoperative course was uneventful, and she was discharged on the 12th postoperative day. To date, the patient is alive, without any evidence of recurrence.

## Discussion

Pancreatic isolated metastases are a rare condition, most frequently originating from primary renal cell carcinomas [8]. The incidence of metastatic pancreatic tumors has been reported to be 3% to 11% [9, 10]. A uterine sarcoma metastasized to the pancreas is an extremely rare condition which ensures that the decision regarding treatment is difficult and nonstandardized. The decision is determined by an attending multidisciplinary oncology team.

Differentiation of a primary pancreatic adenocarcinoma or of neuroendocrine tumors from a metastatic pancreatic tumor is required to perform neoadjuvant therapy. CT and magnetic resonance imaging are used for the evaluation of the pancreatic mass. Highly vascularized tumors are more likely to be metastases than primary tumors, which are hypovascular. The difficulty lies in distinguishing the tumors, on the basis of imaging, between pancreatic metastatic tumors and pancreatic neuroendocrine tumors, which are hypervascular as well [11]. Percutaneous fine-needle aspiration (FNA) is helpful in the preoperative differential diagnosis between a primary and metastatic pancreatic tumor. However, the published data have shown a difference between the immunohistochemical findings on the endoscopic ultrasound-guided FNA (EUS-FNA) and the final immunohistochemistry results from the resected specimen.

The survival benefit of the resection of pancreatic metastases has been demonstrated. Reddy and Wolfgang [12], in a systemic review of the literature, evaluated 243 patients who had undergone radical pancreatic resection for metastatic disease and concluded that the effectiveness of pancreatic resection for a metastatic tumor is dependent on the tumor biology of the primary cancer. In other case reports [3, 5, 6, 10, 13–17], authors have described good long-term survival following an aggressive surgical approach, indicating a possible benefit of surgery in the metastatic setting for selected cases (Tables 1 and 2).

**Table 1:** **First recurrence of pancreatic tumor**

Authors	Primary therapy	Adjuvant therapy	Time to 1st recurrence (months)/site of recurrence	Neoadjuvant treatment	Surgical treatment of 1st recurrence	Adjuvant treatment after 1st recurrence surgery	Time to second recurrence (months)/site of recurrence	Surgical treatment of second recurrence	Postsurgical adjuvant therapy	Survival after pancreatic surgery
Falconi *et al*. [10]	TH + BO (February 1996)	NA	52 (June 2000) Pancreas	Ci + Cy + Do	PPD + LR (March 2001)	No	12 mo (March 2002) liver	TACE	Pelvic recurrence RT and I + D + Da + M (2004)	Alive, 56 mo
Ozturk *et al*. [3]	H + LO + Om (December 2008)	RTh + Do and Ci	Pancreas (May 2013)	No	DP + Spl (HP-leiomyosarcoma)	No	No	No	No	Alive, 6 mo
Kao *et al*. [16]	TH + BO	NA	Lung, pancreas	NA	PD	NA	NA	NA	NA	NA
Skagias *et al*. [15]	2010		Pancreas	no	Partial pancreatectomy

**Table 2:** **Second recurrence of pancreatic tumor**

Authors	Primary therapy	Adjuvant therapy	Time to 1st recurrence (months)/site of recurrence	Neoadjuvant treatment	Surgical treatment of 1st recurrence	Adjuvant treatment after 1st recurrence surgery	Time to second recurrence (mo)/site of recurrence	Surgical treatment of second recurrence	Postsurgical adjuvant therapy	Survival after pancreatic surgery
Iwamoto I et al. [5]	TH + BO (February 2002)	RTh + 18 Gy	12 Lung	No	Video-assisted thoracic surgery	Chemotherapy Ep + Cy + Ca	Pancreas (12 mo)	DP + Spl	No	Alive, 8 mo
Alonso GJ et al. [6]	July 2005	RTh + Br	5 Lung	Chemotherapy	Upper left lobectomy (7 mo)	Chemotherapy (NA)	Pancreas (8 mo)	Segmental pancreatectomy	Recurrence in the lung and liver; bevacizumab (18 mo)	Alive, 18 mo
Hernandez S et al. [14]	H + BO (November 2003	RTh	Lung (2 mo, February 2004)	Ifosfamide + A + Mesna	Segmentary resection	Gemcitabine	Pancreas (47 mo, January 2008	PD (April 2008)	NA	Alive, 67 mo
Clemente G et al. [13]	H + BO	NA	144	NA	PD	NA	NA	NA	NA	NA
Chatzipantelis P et al. [17]	NA	NA	Left axillary and right femoral metastases	NA	NA		Pancreas (FNA) positive for malignancy (120 mo)	DP + Spl		

A, Doxorubicin; BO, Bilateral salpingo-oophorectomy; Br, Brachytherapy; Ca, Carboplatin; Ci, Cisplatin; Cy, Cyclophosphamide; DP + Spl, Distal pancreatectomy and splenectomy; E, Epirubicin; LO, Left oophorectomy; LR, Liver wedge resection; Om, Omentectomy; PH, Partial hysterectomy; PPPD, Pylorus-preserving pancreaticoduodenectomy; Rth, Radiotherapy; SP, Segmental pancreatectomy; TH, Total hysterectomy. FNA-papillary cystadenoma **Fine-needle aspiration = Highly atypical malignant cells, but not conclusive for diagnosis; histopathological diagnosis was uterine leiomyosarcoma with pancreatic metastasis.

In our patient, the long interval between the detection of the pancreatic mass and its resection (33 months), during which time the patient was managed by subsequent cycles of chemotherapy, provided the reason for the oncologist’s and surgeon’s reluctance to resort to surgery, questioning its benefit in a secondary metastatic setting. A wait-and-see attitude regarding chemotherapy was preferred, and the decision to perform surgery was taken as a result of suspected chemoresistance after a long interval of stable disease.

## Conclusions

Surgery can be considered in the pancreas as a unique metastatic site or even in cases with resectable oligometastases. We hypothesize that a long disease-free interval following resection of a primary tumor might be an indicator of a more indolent tumor biology or chemoresponsiveness, and thus we question the possibility of the surgical benefit for these select cases.

## Consent

Written informed consent was obtained from the patient for publication of this Case report. A copy of the written consent is available for review by the Editor-in-Chief of this journal.

## Abbreviations

CT: Computed tomography
FNA: Fine-needle aspiration
LMS: Leiomyosarcoma
PET: Positron emission tomography.

## References

[1] Salazar OM, Bonfiglio TA, Patten SF, Keller BE, Feldstein M, Dunne ME, Rudolph J (1978). Uterine sarcomas: natural history, treatment and prognosis. Cancer.

[2] Salazar OM, Dunne ME (1980). The role of radiation therapy in the management of uterine sarcomas. Int J Radiat Oncol Biol Phys.

[3] Ozturk S, Unver M, Ozturk BK, Bozbıyık O, Erol V, Kebabcı E, Olmez M, Zalluhoglu N, Bayol U (2014). Isolated metastasis of uterine leiomyosarcoma to the pancreas: report of a case and review of the literature. Int J Surg Case Rep.

[4] Rose PG, Piver MS, Tsukada Y, Lau T (1989). Patterns of metastasis in uterine sarcoma: an autopsy study. Cancer.

[5] Iwamoto I, Fujino T, Higashi Y, Tsuji T, Nakamura N, Komokata T, Douchi T (2005). Metastasis of uterine leiomyosarcoma to the pancreas. J Obstet Gynaecol Res.

[6] Alonso Gómez J, Arjona Sánchez A, Martínez Cecilia D, Díaz Nieto R, Roldán de la Rúa J, Valverde Martínez A, Lizárraga Febres E, Padillo Ruiz J, Rufián Peña S (2012). Uterine leiomyosarcoma metastasis to the pancreas: report of a case and review of the literature. J Gastrointest Cancer.

[7] Ogura T, Masuda D, Kurisu Y, Miyamoto Y, Hayashi M, Imoto A, Takii M, Takeuchi T, Inoue T, Tokioka S, Uchiyama K, Umegaki E, Higuchi K (2013). Multiple metastatic leiomyosarcoma of the pancreas: a first case report and review of the literature. Intern Med.

[8] Sweeney JT, Crabtree DK, Yassin R, Somogyi L (2002). Metastatic uterine leiomyosarcoma involving the pancreas diagnosed by EUS with fine-needle aspiration. Gastrointest Endosc.

[9] Adsay NV, Andea A, Basturk O, Kilinc N, Nassar H, Cheng JD (2004). Secondary tumors of the pancreas: an analysis of a surgical and autopsy database and review of the literature. Virchows Arch.

[10] Falconi M, Crippa S, Sargenti M, Capelli P, Pederzoli P (2006). Pancreatic metastasis from leiomyosarcoma of the broad ligament of the uterus. Lancet Oncol.

[11] Z’graggen K, Fernandez-del Castillo C, Rattner DW, Sigala H, Warshaw AL (1998). Metastases to the pancreas and their surgical extirpation. Arch Surg.

[12] Reddy S, Wolfgang CL (2009). The role of surgery in the management of isolated metastases to the pancreas. Lancet Oncol.

[13] Clemente G, Giordano M, De Rose AM, Nuzzo G (2010). Image of the month: metastasis from leiomyosarcoma in the head of the pancreas. Arch Surg.

[14] Hernández S, Martín-Fernández J, Lasa I, Busteros I, García-Moreno F (2010). Pancreaticoduodenectomy for metastasis of uterine leiomyosarcoma to the pancreas. Clin Transl Oncol.

[15] Skagias L, Ioulia E, Politi E (2010). Isolated pancreatic metastasis of uterine leiomyosarcoma diagnosed by fine needle aspiration biopsy. Acta Cytol.

[16] Kao YH, Saad U, Tan AE, Magsombol BM, Padhy AK (2011). Fluorine-18-fluorodeoxyglucose PET/CT for the evaluation of suspected recurrent uterine leiomyosarcomas. Acta Radiol.

[17] Chatzipantelis P, Karvouni E, Fragoulidis GP, Voros D, Pafiti A (2006). Clinicopathologic features of two rare cases of mesenchymal metastatic tumors in the pancreas: review of the literature. Pancreas.

